# Low risk of avian influenza A (H5N6) transmission to depopulation workers in Korea

**DOI:** 10.1111/irv.12530

**Published:** 2018-01-05

**Authors:** Sukhyun Ryu, Jun‐Sik Lim, Benjamin J. Cowling, Byung Chul Chun

**Affiliations:** ^1^ Division of Infectious Disease Control Gyeonggi Provincial Government Suwon Korea; ^2^ Department of Epidemiology and Medical Informatics Graduate School of Public Health Korea University Seoul Korea; ^3^ Disease Diagnostic Team Gyeonggi Province Veterinary Service Suwon Korea; ^4^ WHO Collaborating Centre for Infectious Disease Epidemiology and Control School of Public Health Li Ka Shing Faculty of Medicine University of Hong Kong Hong Kong China; ^5^ Department of Preventive Medicine Korea University College of Medicine Seoul Korea

**Keywords:** avian influenza, personal protective equipment, prevention, transmission

## Abstract

An outbreak of highly pathogenic avian influenza A (H5N6) virus occurred between November 20, 2016, and March 1, 2017 in poultry farms, in the Gyeonggi Province, Republic of Korea. To identify the risk of transmission of H5N6 to depopulation workers, active and passive surveillance was conducted. Virological testing of respiratory swabs with real‐time reverse transcription‐polymerase chain reaction was performed for workers who reported respiratory symptoms. Among 4633 depopulation workers, 22 reported respiratory symptoms, but all tested negative for H5N6. Personal protective equipment in addition to antiviral prophylaxis was adequate to limit transmission of H5N6 from poultry to humans.

## 1 INTRODUCTION

Human infection with HPAI viruses poses an emerging public health threat, because genetic reassortment with human virus could cause a human pandemic.^1^, ^2^ Migratory birds play an important role in the occurrence of avian influenza A viruses in domestic poultry, thus, various subtypes of HPAI have spread in Asian countries including China, Vietnam, Laos, Japan, and Republic of Korea.^3^ Because poultry‐human transmission of HPAI H5 is likely to occur by direct contact with infected poultry,^1^, ^4^, ^5^ early detection and depopulation of infected poultry flocks are commonly used to control the spread of HPAI virus in poultry and to prevent human infections.^6^

Starting November 20, 2016, poultry infections by HPAI (H5N6) virus were reported in Gyeonggi Province, Republic of Korea. A series of control measures, including depopulation, were rapidly implemented by the Korean Ministry of Agriculture, Food and Rural Affairs. Due to the prevailing risk of poultry‐human transmission of HPAI H5N6 virus during depopulation activities, a surveillance program and virological testing were performed to detect the possible human cases from depopulation workers.

Here, we conducted a descriptive study to describe the results from the surveillance of the population directly involved in handling infected poultry.

## 2 MATERIALS AND METHODS

### 2.1 Data collection and case definition

The data regarding depopulation workers were collected during the period between November 20, 2016, (the day of 1st case reported) and April 13, 2017, (the day of declaration of HPAI H5N6 virus outbreak termination). The source “at risk” population of this study was defined as the participant of the depopulation activity who had direct contact with potentially infected poultry. We collected the information of the at‐risk population through questionnaires before the initiation of depopulation activity. All depopulation workers were asked about their age, sex, underlying diseases, type of employment status for a depopulation activity, nationality, and vaccination history of seasonal influenza by the staff from the local department of public health. Furthermore, the duration of the participation of culling operation of each worker was recorded by day. Field epidemiologists from Gyeonggi Provincial Government reviewed all the collected data manually.

We defined as a suspected case, the depopulation worker who presented with any respiratory symptoms (rhinorrhea, cough, or shortness of breath) with or without fever (over 37.8°C) within 10 days following their last exposure to affected poultry. Confirmed cases were defined as a person meeting the criteria of suspected cases and laboratory tests confirmed by identifying HPAI H5N6 virus from clinical samples using respiratory swab.

### 2.2 Preventive and control measures

Depopulation workers were provided with personal protective equipment (PPE), including disposable coveralls, nitrile gloves, N95 particulate half‐mask with two‐strap design, unvented goggles, and boots. Preventive antiviral prophylaxis with oseltamivir (75 mg once daily) was also administered from the day of the first exposure to the 7 days after the last exposure. Furthermore, to avoid co‐infection with seasonal influenza, trivalent inactivated seasonal influenza vaccines were administered to those who had not already been vaccinated. The local department of public health monitored all workers every day during the culling operation and conducted telephone survey on every fifth and tenth day of their last exposure. Furthermore, the workers were asked to report immediately to the local department of public health when any respiratory symptoms were developed.

### 2.3 Clinical sample collection

The respiratory specimens of individuals with suspected infection were immediately collected by nasopharyngeal swab, when they reported. The delay between symptom onset and the time of sample collection from the cases was measured as well. These samples were kept at 4°C and transported to the provincial public health laboratory. All the respiratory samples were tested using Real‐time Reverse Transcription‐Polymerase Chain Reaction (RRT‐PCR) testing, owing to its high sensitivity in influenza A H5 detection, and other respiratory viruses.^7^

## 3 RESULTS

**Figure 1:**
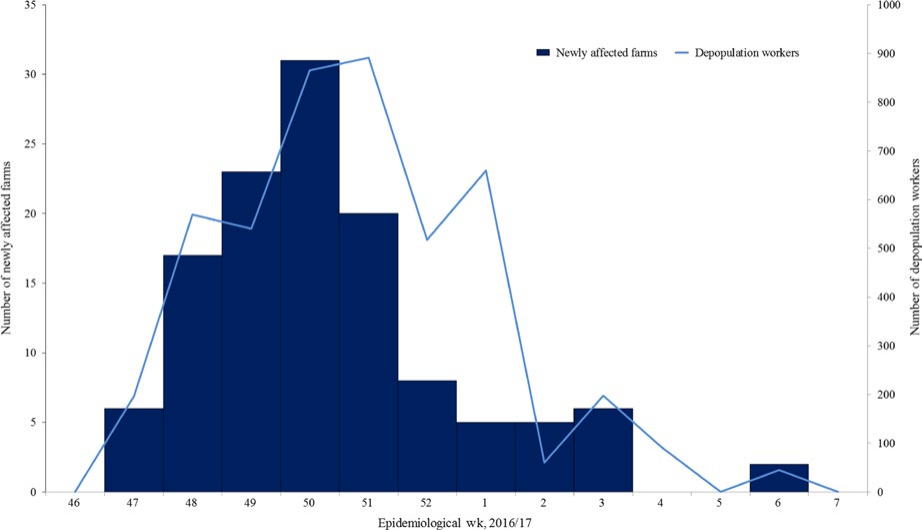
The number of affected poultry farms and the number of depopulation workers during the avian influenza epizootic outbreak in Gyeonggi Province, Republic of Korea by epidemiological weeks from 46 of 2016 to 7 of 2017. Bars show the number of HPAI H5N6 affected poultry farms. Line indicates the number of depopulation workers

From November 20, 2016, to March 17, 2017, 123 affected farms by HPAI H5N6 virus were recorded and 4633 depopulation workers were participated in the study (Figure 1); among them, 4436 (95.7%) were male and 197 (4.3%) were female (Table 1). Fifty‐eight (1.2% of workers) had underlying diseases including cardiovascular (0.7%), endocrine (0.4%), pulmonary (0.1%), and neurologic disease (0.0%). One thousand two hundred and five (26.0% of total) were 50‐59 years old, 1018 (22.0%) were 40‐49, 975 (21.0%) were 30‐39, and 922 (20.0%) were 20‐29. As a type of employment status, 3968 (85.6%) were temporarily contracted as civilians and 665 (14.4%) were public officers. Based on nationality, 2601 (56.1%) were Koreans, 1588 (34.3%) were Asians (non‐Korean), and 277 (6.0%) were Europeans. Two thousand one hundred and ninety‐eight (47.4%) were vaccinated before the outbreak and 2433 (52.5%) were vaccinated during the outbreak. The mean days of participation of depopulation workers were 1.3 days (range: 1‐15 days, total: 19 921.9 days) and the total period of follow‐up was 60 229 days. Twenty‐two (0.5% of total) depopulation workers had ≥1 symptom: 73% had cough, 23% had rhinorrhea, 18% had sore throat, 18% had sputum, 14% had myalgia, and 9% had headache. The mean delay between the onset of symptom and respiratory specimen collection was 108 minutes (range 30‐300 minutes). One of the specimens was identified as a Rhinovirus, and all were confirmed as negative for H5N6 by RRT‐PCR.

**Table 1:** Characteristics of depopulation workers and suspected cases attended to the depopulation campaign during the avian influenza A H5N6 epizootic outbreaks in Gyeonggi Province, Korea

	Depopulation workers (n = 4633)	Suspected cases^a^ (n = 22)
Sex		
Male	4436 (95.7%)	22 (100%)
Female	197 (4.3%)	0
Underlying diseases		
Cardiovascular diseases	33 (0.7%)	0
Endocrine diseases	18 (0.4%)	0
Pulmonary diseases	6 (0.1%)	0
Neurologic diseases	1 (0.0%)	0
Age groups, years		
10‐19	9 (0.2%)	0
20‐29	922 (20.0%)	0
30‐39	975 (21.0%)	7 (31.8%)
40‐49	1018 (22.0%)	8 (36.4%)
50‐59	1205 (26.0%)	5 (22.7%)
60‐69	439 (9.5%)	2 (9.1%)
70‐79	17 (0.4%)	0
>80	5 (0.0%)	0
Unknown	43 (1.0%)	0
Vocation		
Public officers	665 (14.4%)	2 (9.0%)
Civilians (temporary contracted)	3968 (85.6%)	20 (91.0%)
Nationality		
Koreans	2601 (56.1%)	21 (95.5%)
Asians (non‐Koreans)	1588 (34.3%)	1 (4.5%)
Europeans	277 (6.0%)	0
Unknown	83 (1.8%)	0
Middle‐Eastern	76 (1.6%)	0
Africans	8 (0.2%)	0
Seasonal influenza vaccine		
Vaccinated before outbreak	2198 (47.4%)	9 (40.9%)
Vaccinated during outbreak	2433 (52.5%)	13 (59.1%)
Unknown	2 (0.0%)	0

a Suspected cases are the depopulation workers who presented with any respiratory symptoms with or without fever (over 37.8°C) within 10 days following their last exposure to affected poultry.

## 4 DISCUSSION

We evaluated the infection status of workers who directly handled infected poultry, all of whom preventive measures were undertaken. The genetic analysis of collected HPAI A H5N6 virus from Gyeonggi Province revealed that it was highly similar with the virus obtained from Guangdong province in China where human case infected with HPAI H5N6 has been reported since April 2014. It is well established that direct or indirect exposure to infected poultry or contaminated environments such as handling of carcasses increases the risk for human infection by HPAI virus.^8^, ^9^, ^10^ Despite the observation of suspected cases which had more sensitive definition than that of influenza‐like illness, we did not identify any human transmission case among depopulation workers in this study, and this is in agreement with a previous literature.^11^ During the period of outbreak, influenza A H3N2 was the major subtype of seasonal influenza virus, which was relevant to the vaccine administrated to the workers; post‐exposure antiviral prophylaxis with PPE may have limited the chances of infection on depopulation workers. Furthermore, the depopulation method used in this study involved asphyxiating poultry in containers, with using carbon monoxide or carbon dioxide. This culling method may help to limit poultry‐human transmission as well, because the risk is considered relatively low (5.5%), compared with electrocution (8.3%) or injection (20.8%).^12^ Thus, the overall risk of HPAI A H5N6 transmission to depopulation workers in our study was low.

Previous studies indicate that timely and effective prophylaxis in infected poultry minimizes the possibility of human infections.^13^, ^14^ However, the evidence of transmission to depopulation workers, particularly with preventive measures, is still limited.^15^ Thus, a surveillance program for humans at risk is crucial to evaluate and prevent human transmission in the event of an HPAI virus epizootic outbreak.

Our study has several limitations. First, during the post‐exposure monitoring period of depopulation workers, the interview was conducted by telephone. This may have led to under‐reporting of potential cases, because most workers were temporarily employed and reporting a sickness may have led to employment termination. However, there was no additional notice from the regional medical institution where the surveillance program had been operated about any chances of infection related to poultry exposure. Second, there may have been a communication gap due to the language barrier with some foreign workers. Third, we did not conduct the serologic study, which may have increased chances of identifying some infections on depopulation workers. However, the specimens from suspected cases were collected immediately after chances of infection were reported, considering the viral load was relatively high enough to be detected by the RRT‐PCR assay, which is more sensitive than antigen‐capture ELISA and more effective than virus isolation.^7^

## 5 CONCLUSIONS

Poultry‐to‐human transmission of HPAI H5N6 virus was not identified, suggesting that preventive control measures, including PPE and chemoprophylaxis, and depopulation method may have contributed an apparently decreased risk of human transmission.

## DISCLAIMERS

The opinions expressed by authors contributing this journal do not necessarily reflect the opinions of the Gyeonggi Provincial Government.

## CONSENT OF PARTICIPATE

A written informed consent was obtained from the study participants.

## CONFLICT OF INTEREST

The authors declare no conflict of interest.
